# Supplementary figures and images for: Genome-wide scan for selection signatures in Mexican Sardo Negro Zebu cattle
Source: PLoS One. 2024 Nov 11;19(11):e0312453. doi: 10.1371/journal.pone.0312453 (PMC11554216; doi:10.1371/journal.pone.0312453)

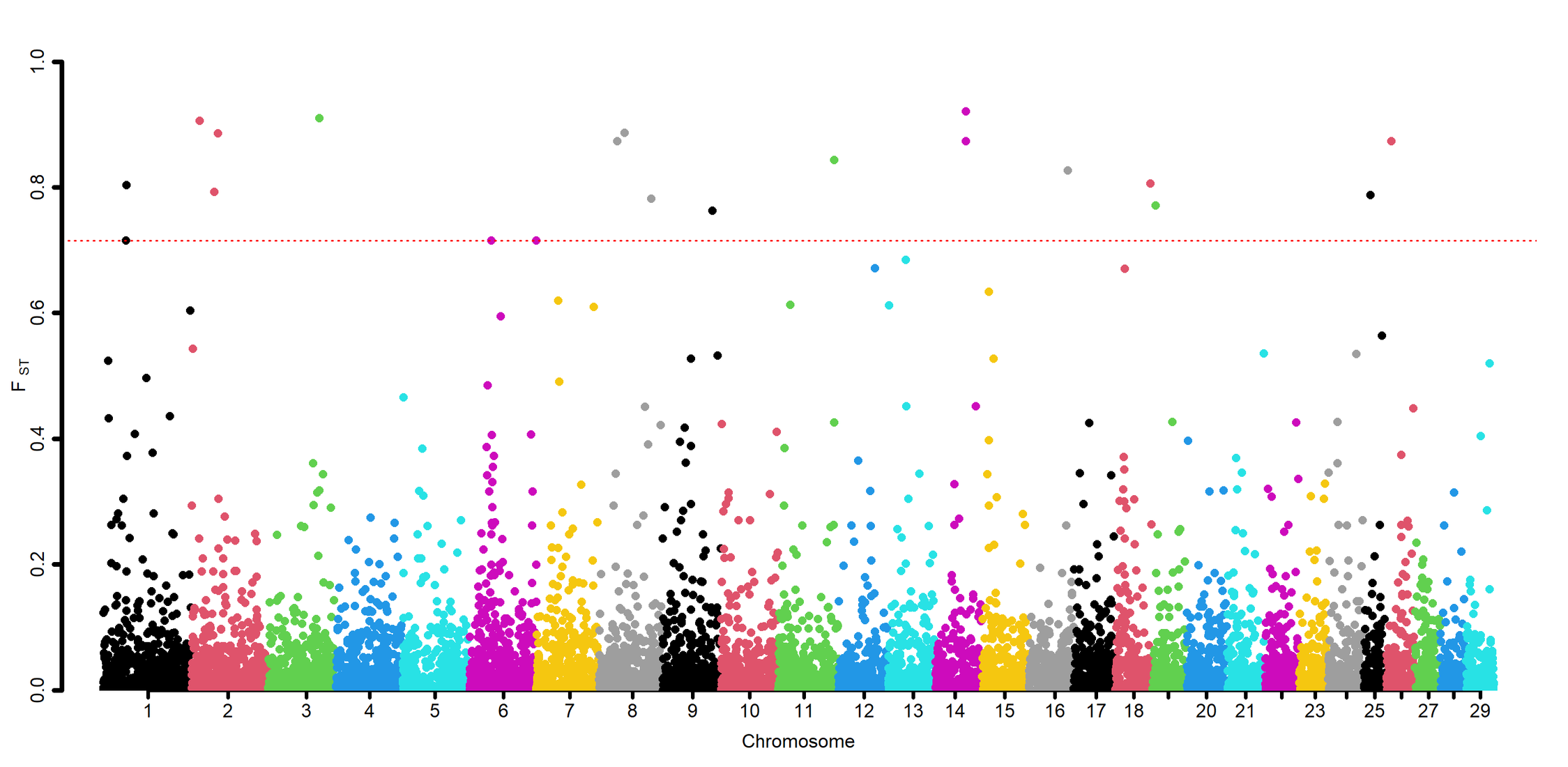

Supplement: S1 Fig — The horizontal line represents the threshold level of 0.1%. (TIF) [file pone.0312453.s001.tif]

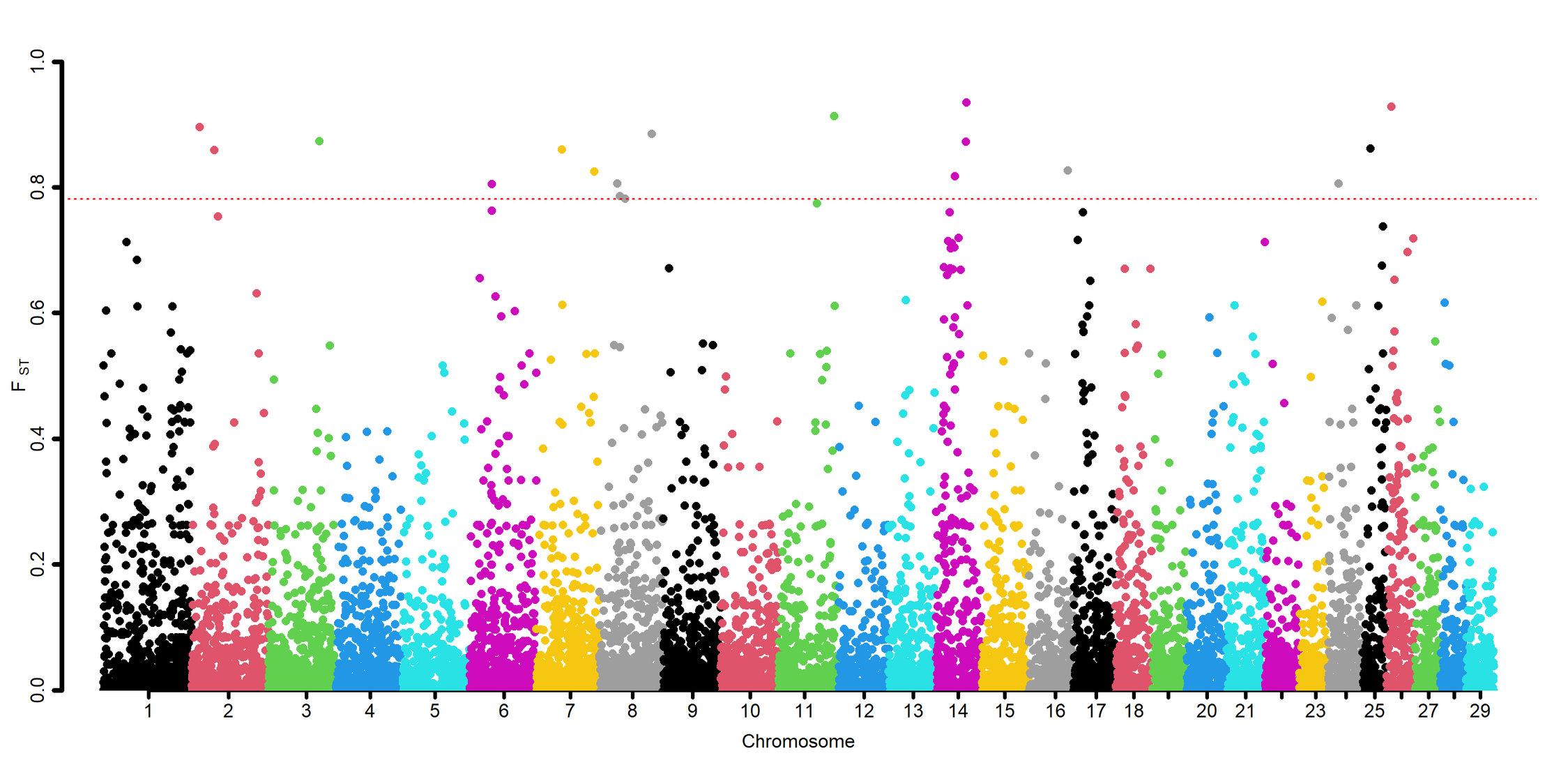

Supplement: S2 Fig — The horizontal line represents the threshold level of 0.1%. (TIF) [file pone.0312453.s002.tif]

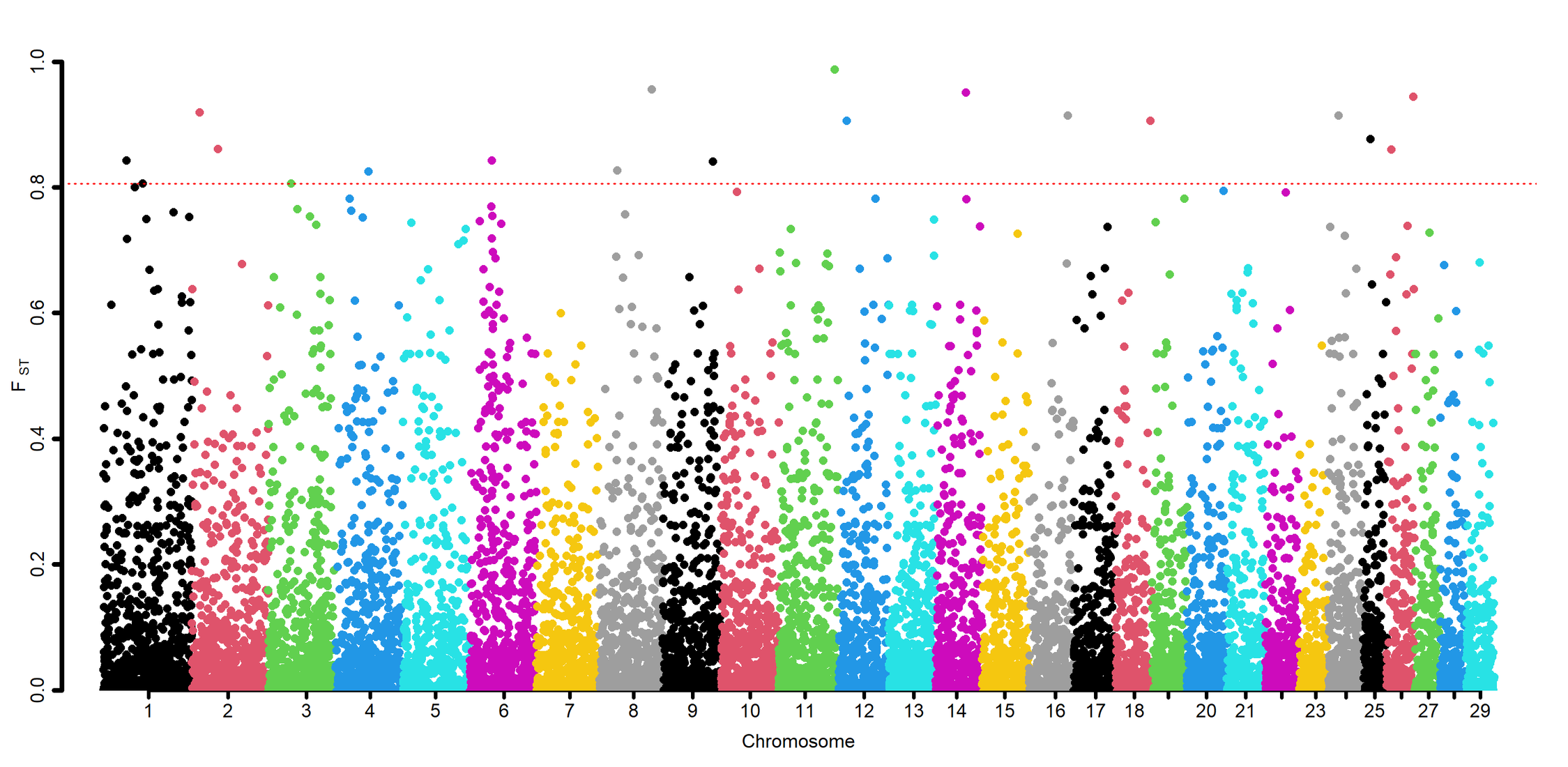

Supplement: S3 Fig — The horizontal line represents the threshold level of 0.1%. (TIF) [file pone.0312453.s003.tif]

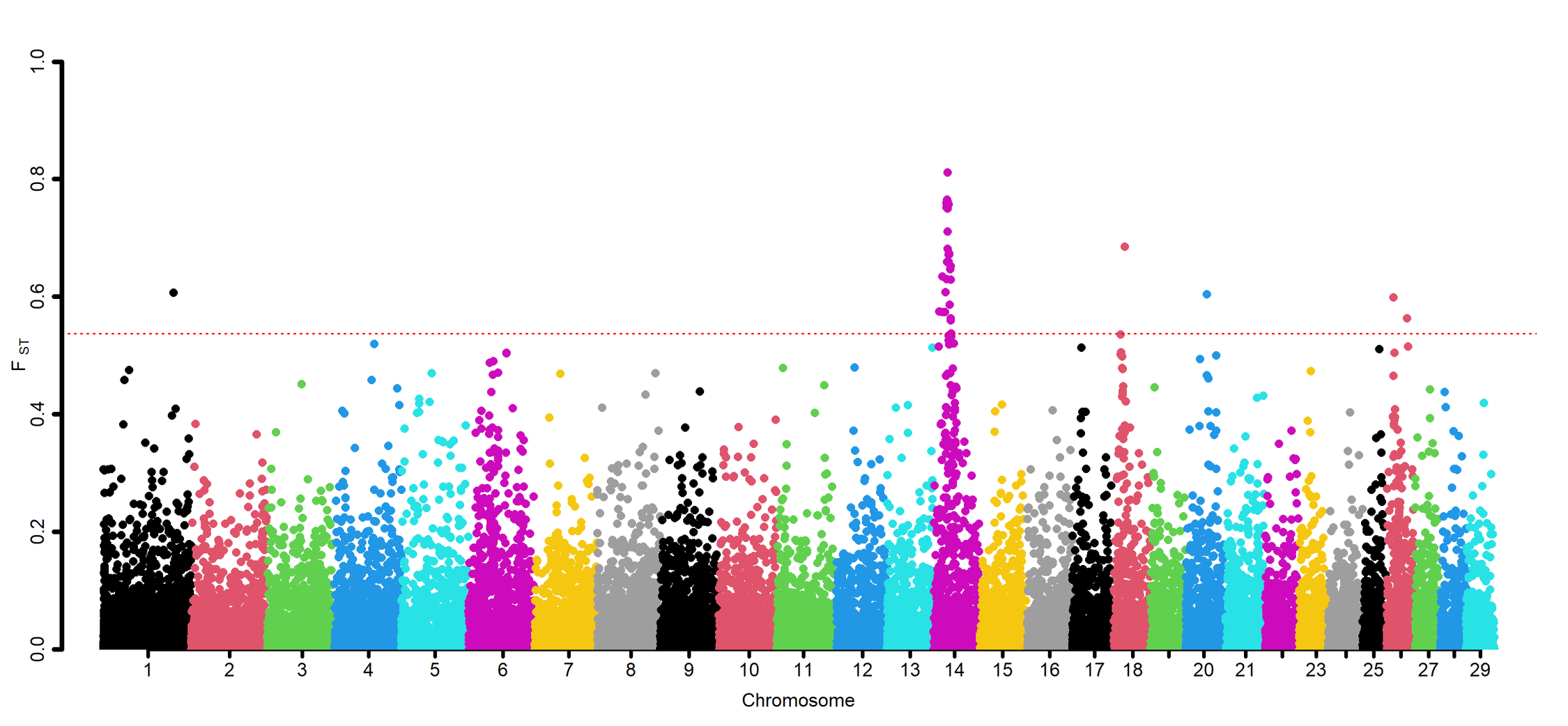

Supplement: S4 Fig — The horizontal line represents the threshold level of 0.1%. (TIF) [file pone.0312453.s004.tif]

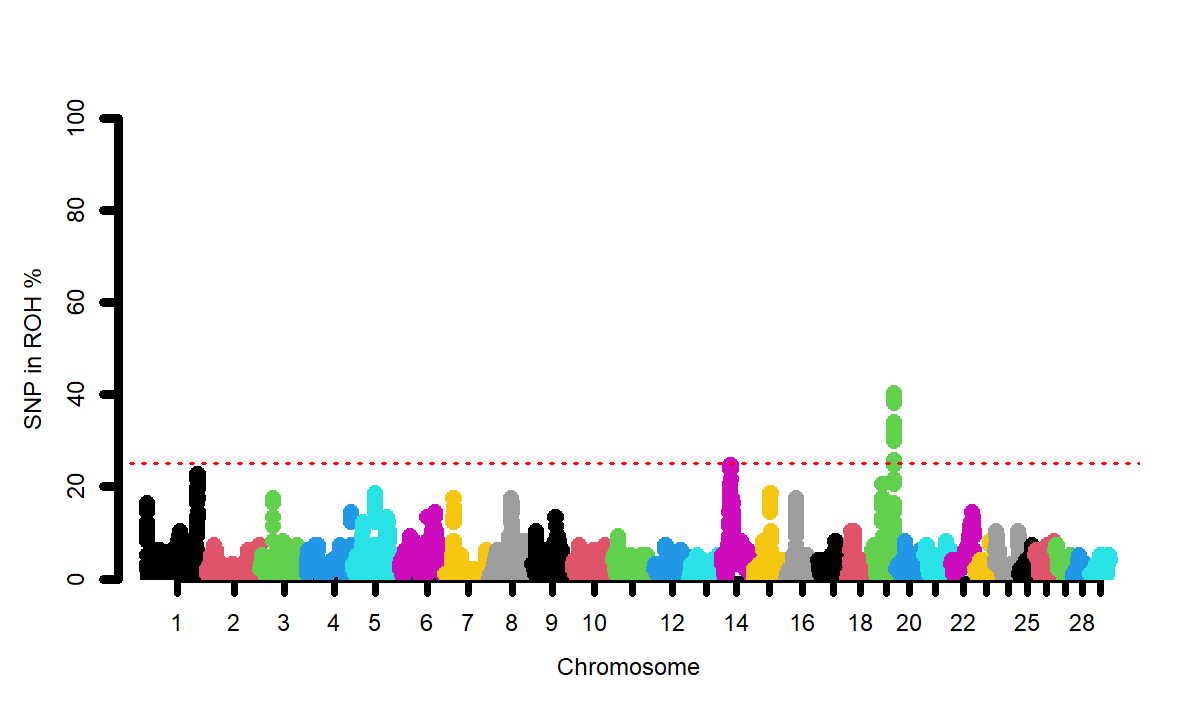

Supplement: S5 Fig — The horizontal dashed line represents the threshold level of 0.1%. (TIF) [file pone.0312453.s005.tif]

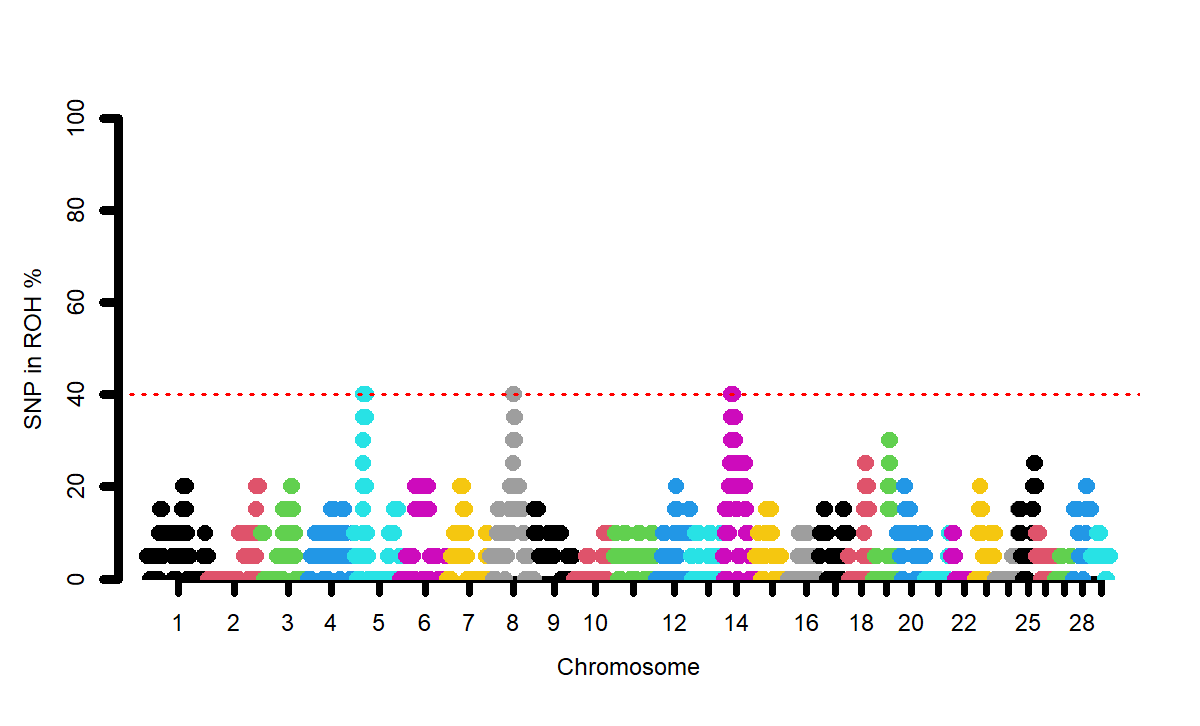

Supplement: S6 Fig — The horizontal dashed line represents the threshold level of 0.1%. (TIF) [file pone.0312453.s006.tif]

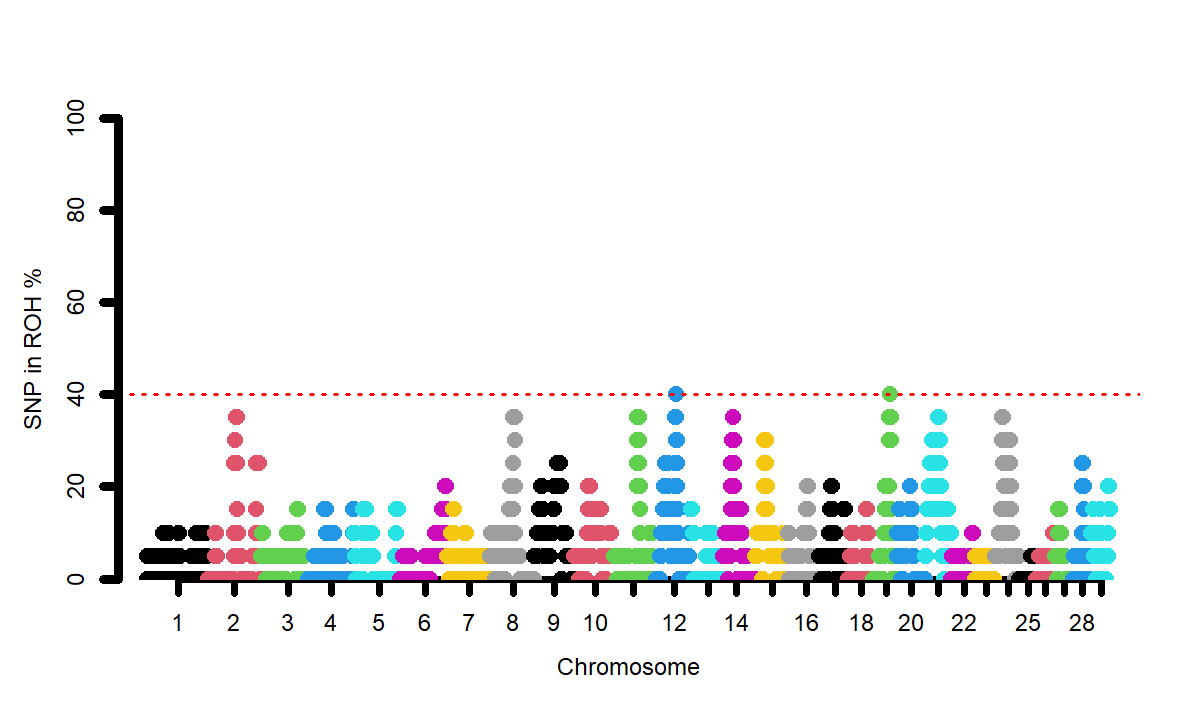

Supplement: S7 Fig — The horizontal dashed line represents the threshold level of 0.1%. (TIF) [file pone.0312453.s007.tif]

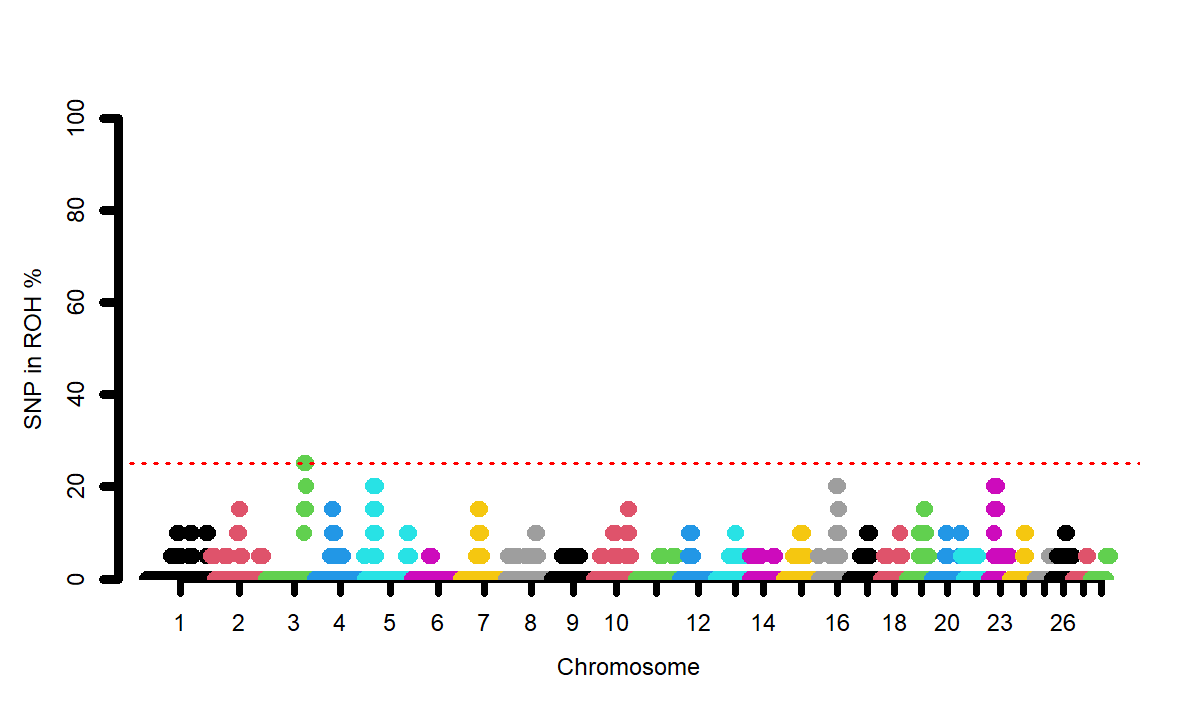

Supplement: S8 Fig — The horizontal dashed line represents the threshold level of 0.1%. (TIF) [file pone.0312453.s008.tif]
